# Immune Persistence After Infant Hepatitis-B Vaccination: A Systematic Review and Meta-Analysis

**DOI:** 10.1038/s41598-018-30512-8

**Published:** 2018-08-22

**Authors:** Sajid Mahmood, Kifayat Ullah Shah, Tahir Mehmood Khan

**Affiliations:** 1 Faculty of Pharmacy, Quaid-e-Azam University, Islamabad, 45320 Pakistan; 2 School of Pharmacy, Monash University, Bandar Sunway, 45700 Selangor, Malaysia; 3 The Institute of Pharmaceutical Sciences (IPS), University of Veterinary & Animal Sciences (UVAS), Outfall road, Lahore, Pakistan

## Abstract

A systematic review was performed to estimate the duration of protection of Hepatitis-B vaccine after primary vaccination during infancy. The number of seropositive participants with anti-HBs antibody titer ≥ 10 mIU/ml and seronegative participants who had anti-HBs antibody titer ≤ 10 mIU/ml after booster dose was the main outcome criteria to find out the protection time of Hepatitis-B vaccine. Twelve studies were selected for systematic review. Overall, results from the meta-analysis have revealed that the risk of Anti-HBs Titer ≤ 10 mIU/ml reduced by 50%. Upon performing the sub-group analysis it was revealed that the overall risk of having Anti-HBs Titre ≤ 10 mIU/ml was reduced up to 62% among the subjects age 21–30 years (0.38 [0.34, 0.44]; I^2^ = 0.0%, p = 0.938). Furthermore, it was observed that the risk of having titre level less than 10 mIU/ml for plasma derived vaccines were to be 56% [0.44, CI 0.33–0.57, I^2^ 90.9%, p = <0.001]. Vaccination in early infancy does not ensure protection against Hepatitis-B infection. There is a strong correlation between the duration of protection and time elapsed after primary immunization during infancy.

## Introduction

Hepatitis-B is a serious health problem worldwide. According to World Health Organization (WHO) in year 2016 about 240 million people were infected with Hepatitis-B^1^, and about 686,000 death were reported^2,3^. Countries with the highest disease burden are China, Indonesia, Nigeria, parts of Africa and Asia^4,5^. Norway and United Kingdom are observed to be the countries with the prevalence of hepatitis-B, as low as 0.01%, while the highest prevalence is observed in Sudan where it is up to 22.70%^6^.

In South Asia the prevalence rate of chronic Hepatitis-B is 2–5%^1^. A decrease in hepatitis-B prevalence has been seen in countries where routine immunization plan have been implemented^7^.

Vaccination with Hepatitis-B has been considered as a very important tool for protection against HBV infection^8^. The protective response to Hepatitis-B vaccine is quantified by measuring anti-HBS level in 6–8 weeks after vaccination, for successful immunization the anti-HBS level should be greater than 10 mIU/ml. According to WHO the Hepatitis-B vaccination should produce the protective level of antibodies in ≥95% of the individuals after completion of the recommended vaccination schedule^9^. However, in some cases primary and secondary vaccination failure led to occurrence of hepatitis B infection among the individual. When the Infections occur in short time after the vaccination, it is termed as primary vaccination failures. However, in the case when there is loss of seroprotective response that is termed as secondary vaccination failure due to loss of immunity^10^, which is due to decline in the immunological memory which wane over time^11^. The individual whose anti-HBS level falls below 10 mIU/ml is not protected anymore. However the individual is not under the threat from hepatic disease because of immune memory related to Hepatitis-B surface antigen (HBsAg). The specific memory after Hepatitis-B vaccination is due to an anamnestic anti-HBs response after booster dose of vaccine. The booster dose lead to spontaneous rise in anti-HBs level in the population who have completed their initial vaccination series^12,13^. According to European consensus group on Hepatitis-B immunity the duration of protection among fully vaccinated children is 15 years^12^.

The protection period of Hepatitis-B vaccine (either derived from plasma or recombinant vaccine) is not well understood^8,14–16^. According to WHO global immunization coverage data sheet 2014 highest coverage for Hepatitis-B vaccine is seen in western pacific where it is estimated to be 92%,while lowest is 10% in African region.

## Study Question and Aim

The duration of protection of Hepatitis-B vaccine is still variable and a very limited data is available on this topic especially in developing countries. Most regions/countries have different vaccination schedule, this results in failure to develop the policies and procedures to control the spread of Hepatitis-B in the world, particularly in under developed countries. Currently available vaccines are highly safe and effective but the available data shows that antibody titer declines with time^17,18^. Secondary vaccination failure is one of the main concern for the most of the policy makers in order to ensure the decline in mortality and morbidity of.

Therefore, this systematic review aims at assessing the duration of protection of Hepatitis-B vaccine after primary vaccination during infancy/childhood as well as the need for booster dose in case the antibody titer is below immunoprotection level. Findings in this regard will assist the policy makers to develop guidelines for booster dose.

## Method

A systematic review of the scientific literature was performed. All studies published from 1^st^ Jan 2000 till 31^st^ December 2016 was assessed for potential inclusion in this systematic review.

### Search Strategy

The syntax used for literature survey is Immune Memory OR immunopersistence AND Hepatitis-B AND Vaccine OR vaccination OR Immunization AND infants OR newborn OR birth OR cohort. The relevant studies were identified through Pubmed, Medline, Embase, Google Scholar and Cochrane Library.

**Population, intervention, comparator and outcomes**

**Population = **Seronegative children/adults

**Intervention = **Hepatitis-B vaccine

**Comparator = **None

**Outcome = **The outcomes studied in this systematic review include the percentage of the children/adults with antibody titer ≥10 mIU/ml and percentage of seronegative children/adults who have seroprotective antibody titer ≥10 mIU/ml in response to single booster dose. In addition, continuous data i.e. antibody titer, standard deviation and number patients before and after the booster dose will also be extracted to estimate the differences in the titer before and after vaccination.

### Inclusion Exclusion Criteria

All observational studies reporting the antibody titer after primary vaccination with Hepatitis-B vaccine during infancy with or without the information regarding the administration of booster dose were included for further assessment.

We excluded all case studies/reports, letter to the editors, review papers, personal opinions or any other type of study with inconsistent data or not reporting original data. Similarly the studies were excluded if primary vaccination was done six months after birth. All studies with follow up duration less than two years were also excluded. Studies were excluded if the booster dose was administered between the primary vaccination series and follow up study.

### Data extraction

The titles, abstracts and the contents of the study were reviewed according to our inclusion and exclusion criteria. Any question/concern regarding the selection of articles was settled through consensus. The data collected was Name of investigator, Country of study, Year of study, Study population, No. of patients with Hepatitis-B antibody titer ≥ 10 mIU/ml, No. of patients with Hepatitis-B antibody titer ≥ 10 mIU/ml after booster dose, Vaccine type, Dosage of vaccine and Dosage schedule. Two investigators independently evaluated the identified research studies and extracted the relevant outcomes of the studies. The outcomes of the studies were extracted by using the data extraction table especially designed for this purpose (Table 1). Any confusion regarding the results of selected study was resolved through mutual consensus.

**Table 1:** Anti-HBs Titer ≥ 10 mIU/ml before and after Booster Dose.

Authors	Country of Study	Study Design	Year of Study	Age at Follow up (Years)	Study Population	Patients with anti-HBS ≥ 10 mIU/ml	Anti-HBS antibody Titer After Boosting	Vaccine Type	Dosage of Vaccine	Vaccination Schedule 1 ^s^ 2^nd^ 3^rd^		
Bruce *et al*.	Alaska	Prospective Cohort	2011	30	243	51%	88%	Plasma Derived	Standard	Vaccinated at 06 months age.		
Wu *et al*.	China	Randomized placebo controlled cohort	2010	23	126	48.1%	84%	Plasma Derived	Standard	00	01	06
Poovorawan *et al*.	Thailand	Cohort Study	2009	20	109	60.5%	83.9%	Recombinant	Standard	00	01/02	12
Al-Ghamdi *et al*.	Saudi Arabia	Cross Sectional Cohort Study	2012	20	238	58.8%	Not Specified		Standard			
Su *et al*.	Taiwan	Cross Sectional Study	2012	18	1734	35.9%	95%	Plasma Derived	Standard	01	02	12
Al Faleh *et al*.	Saudi Arabia	Cross Sectional Study	2008	18	1355	38.0%	Not Specified	Recombinant	Standard	00	01	06
Spada *et al*.	Italy	Follow up Study	2010	17	571	72.9%	Not Specified		Standard			
Middleman *et al*.	U.S.A	Cross Sectional Study	2014	16–19	420	24.0%	92%	Recombinant	Standard	00	01	12
Avidoca *et al*.	Slovakia	Longitudinal Study	2010	10–11	31	48.4%	96%		Standard	03	05	12
Gold *et al*.	Israel	Cross Sectional Study	2000	08	122	77.1%	Not Specified	Recombinant	Standard	00	01	06
Tsega *et al*.	Ethiopia	Longitudinal Study	2010	05	314	89%	Not Specified	Recombinant	Standard	00	01	06
Amini *et al*.	Iran	Longitudinal Study	1999	02	542	97% (Children) 82% (Adults)	Not Specified	Recombinant	Standard	00	01	06

### Data analysis

Meta-analysis was carried out by using STATA version 14.3®. Risk ratios were estimated using random effect model. The binary data was analyzed to estimate the risk of having Hepatitis-B antibody titer ≥ 10 mIU/ml after booster dose. Subgroup analysis was performed to address heterogeneity. Age and type of vaccines were used as the grouping variables. Tau^2^ was used to interpret the heterogeneity at a confidence interval of 95%. In addition, quality of studies was also performed using New Castel Ottawa scale for observational studies.

## Results

**Figure 1:**
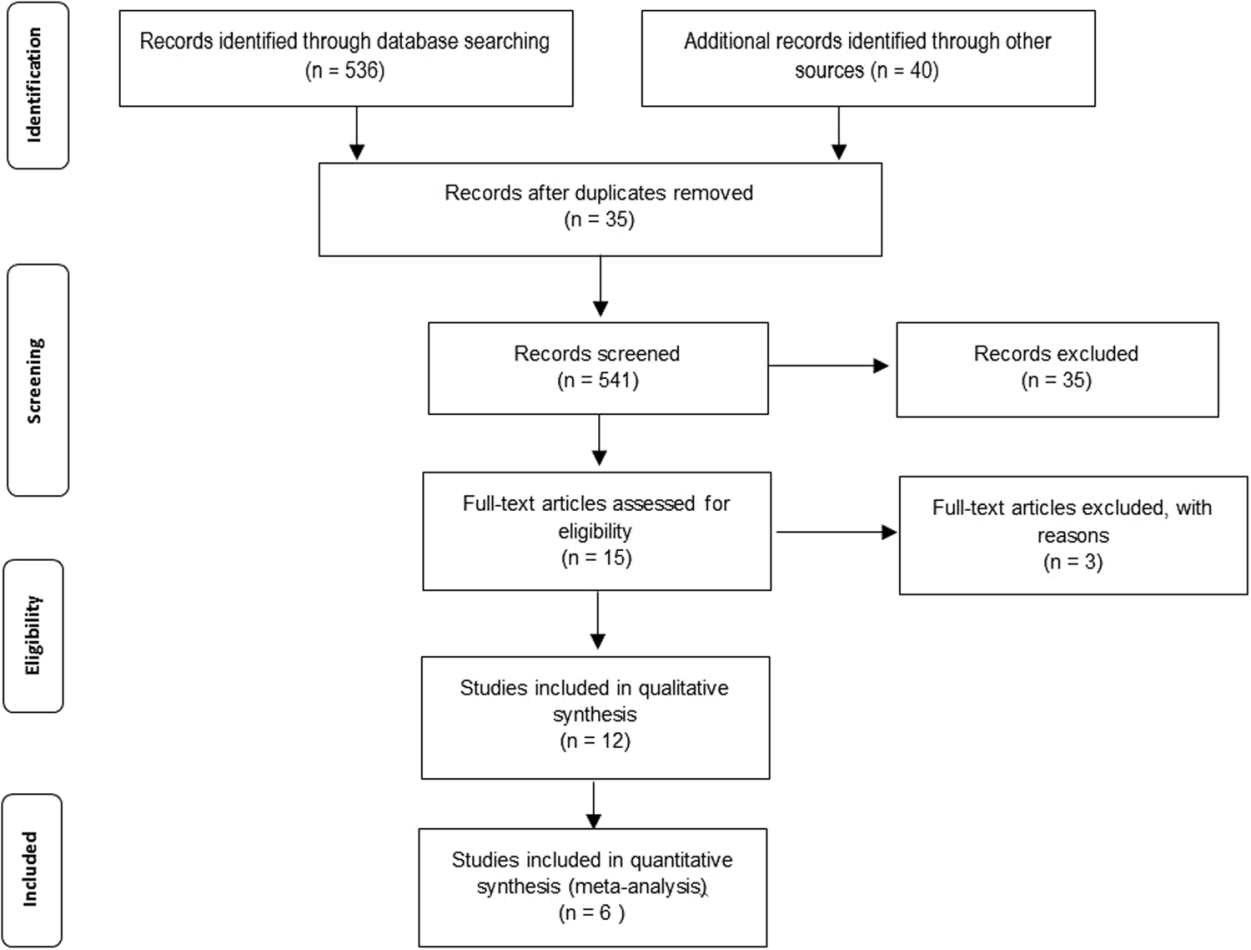
PRISMA 2009 Flow Diagram.

A total of N = 576 studies were identified for the initial assessment and potential inclusion in this systematic review. Upon applying the inclusion criteria n = 12 studies were eligible for the qualitative synthesis. Upon screening the extracted data, n = 6 studies were selected for the meta-analysis. Details illustration of the screening the selected articles are shown in the Fig. 1.

### Persistence of seroprotective Anti-HBs Titer

A total of N = 5805 patients were presented across the twelve studies^8,19–29^. Only six studies have reported the anti HBS titer after booster dose of hepatitis-B vaccine, with a follow up of two years^8,19,20,22,25,26^. A single booster dose of vaccine was administered only to those participants who had antibody titer ≤ 10 mIU/ml at the time of follow up, antibody titer was again determined one month after challenge dose. However Su *et al*. administered two subsequent booster doses of Hepatitis-B vaccine. Second booster dose was administered six months after the first dose, if the participant had ant-HBV titer ≤ 10 mIU/ml after the first challenge dose. Anti-HBV antibody titer was again determined one month after the second challenge dose^22^. An increase in anti HBS titer ≥ 10 mIU/ml after booster dose ranging from 23.4% to 68.0% of participants than the anti HBS titer before booster dose. Details are given in Table 1. Among the eligible studies one was randomized placebo controlled study^19^, one study was Phase-4 Open follow up and Challenge Study^26^, three were Cohort Studies^8,20,21^. Three were follow up studies^24,28,29^ four were cross sectional studies^22,23,25,27^.

All participants of the study were fully immunized during infancy with either recombinant or plasma derived hepatitis-B vaccine. Bruce *et al*. in a long term prospective cohort study conducted in Alaska, data was collected after thirty years of the primary vaccination with plasma derived vaccine on 243 participants. Of whom 51% of the participants has anti-HBs titer above seroprotection level (titer ≥ 10 mIU/ml)^8^. In a randomized placebo controlled trial cohort study conducted in China on 126 persons immunized with plasma derived vaccine during infancy it was observed that 48.1% of subjects were seroprotective with an antibody titer ≥ 10 mIU/ml after 23 years^19^. In a cohort study conducted in Thailand by Poovorawan *et al*. reported data of 109 participants with recombinant Hepatitis-B vaccine, of whom 60.5% of participants were maintained with the seroprotective level of Anti-HBs anti-bodies^20^. Al-Ghamidi *et al*. reported data of 238 participants from a cross sectional study conducted in Saudi Arabia, it was observed that 58.8% participants were seroprotected after twenty years after primary vaccination with recombinant hepatitis-B vaccine^21^. During an eighteen years cross sectional study in Taiwan on 1734 participants, only 35.9% of participants were seroprotected^22^. In eighteen years cross sectional cohort study in Saudi Arabia by Al-Faleh *et al*. on 1355 participants, only 38% of the participant had seroprotective anti-HBs tier ≥ 10 mIU/ml^23^. Spada *et al*. conducted a 17 years follow up study on 571 participants in Italy and noticed that 72.9% of participants were seroprotected^24^. In a recent cross sectional study conducted by Middleman *et al*. in USA among 420 Participants, of whom only 24% of the participants were seroprotected, 16–19 years after primary vaccination with Recombinant Hepatitis-B vaccine during infancy^25^. In an eleven years phase-4 open follow up and challenge study on 31 participants in Slovakia by Avidoca *et al*., the seroprotection level was to be 48.4%^26^. Gold *et al*. in a cross sectional study in Israel found that 77.1% of the participants were seropotected^27^. Tsega *et al*. in a follow up study carried out five years after primary vaccination with recombinant Hepatitis-B vaccine in Ethiopia revealed that 89% of participants were still seroprotected^28^. In a two years follow up study conducted by Amini *et al*. in Iran on 542 participants establish that 97% of the participants had anti-HBs titer ≥ 10 mIU/ml after primary immunization with recombinant Hepatitis-B vaccine during infancy^29^.

### Response to Booster Dose

The antibody titer ≤ 10 mIU/ml does not always means the loss of immunity because the vaccine protection persists beyond the time during which antibody titer is above seroprotective level. The rise in anti-HBs titer above seroprotection level after the booster dose means that anamnestic response to vaccine still persists^30^. For example, results Bruce *et al*. revealed that Anti-HBs antibody titer above seroprotection level raised from 51% to 88% after booster dose which shows that immune memory still persists even though the Anti-HBs level is below seroprotection level^8^. Similarly in the randomized placebo controlled trial cohort study carried out by Qian Wu *et al*. the number of seroprotected individuals rise from 48.1% to 84% after booster dose^19^. Poovorawan *et al*. in a cohort study in Thailand observed that number of seroprotected individuals rose from 60.5% to 83.9% after a single booster dose^20^. Su *et al*. in across sectional study in Taiwan observed a rise in Anti-HBs level above seroprotection level from 35.9% to 95% after booster dose which shows the persistence of immune memory^22^. Middleman *et al*. in a cross sectional study conducted in USA observed an increase in the number of seroprotected individuals from 24% to 92% after single booster dose^25^. Avidoca *et al*. in a phase-4 open follow up and challenge study in Slovakia observed that the number of seroprotected individuals rose from 48.4% to 96% showing the persistence of immune memory Eleven years after primary vaccination during infancy^26^.

### Quality of studies

All the studies included in this systematic review were observational studies. New castle Ottawa scale was used to estimate the quality. Overall most studies were of good quality with a NOS score ranging from 7 to 8. Details are shown in Table 2.

**Table 2:** Quality assessment using New Castel Ottawa Scale.

Authors/reference	Selection				Comparability	Outcomes			Total Score
	Representative of Exposed Cohort	Selection of Non-Exposed Cohort	Ascertainment of Exposure	Outcomes not Present at Baseline		Assessment of Outcomes	Sufficient Follow up Duration	Adequate Follow up	
Bruce *et al*.	1	0	1	1	2	1	1	1	8
Wu *et al*.	1	0	1	1	1	1	1	1	7
Poovorawan *et al*.	1	0	1	1	2	1	1	1	8
Al-Ghamdi *et al*.	1	0	1	1	1	1	1	1	7
Su *et al*.	1	0	1	1	1	1	1	1	7
Al Faleh *et al*.	1	0	1	1	1	1	1	1	7
Spada *et al*.	1	0	1	1	2	1	1	1	8
Middleman *et al*.	1	0	1	1	2	1	1	1	8
Avidoca *et al*.	1	0	1	1	2	1	1	1	8
Gold *et al*.	1	0	1	1	2	1	1	1	8
Tsega *et al*.	1	0	1	1	1	1	1	1	7
Amini *et al*.	1	0	1	1	1	1	1	1	7

### Meta-analysis for the post booster titer comparison

**Figure 2:**
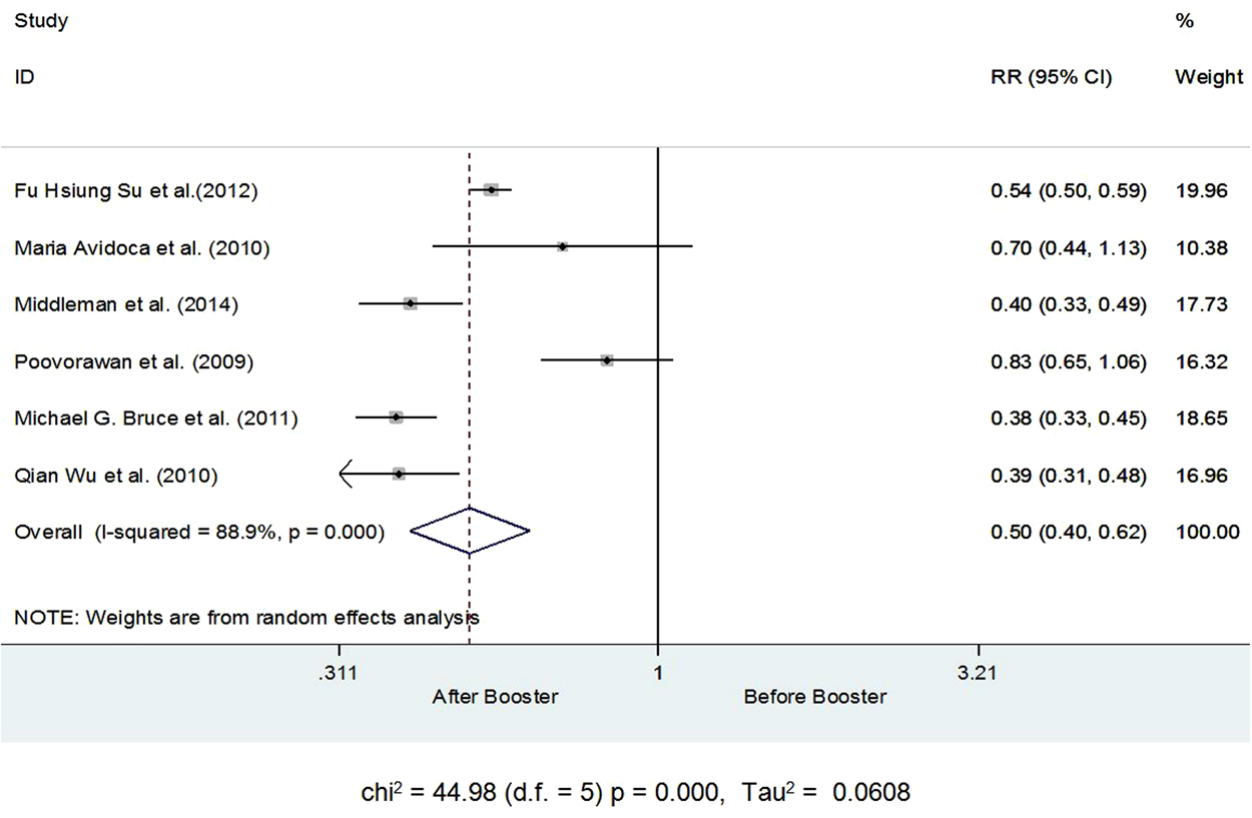
Number of subjects with Anti-HBs Titer ≥ 10 mIU/ml before and after the booster dose.

Quantitative data from six studies were considered suitable for the Meta-analysis. Number of subjects with Anti-HBs Titer ≥ 10 mIU/ml before and after the booster dose was used to estimate the overall effect of the booster dose. Upon initial analysis it was revealed that after receiving the booster dose the risk of having Anti-HBs Titer ≤ 10 mIU/ml reduced by 50% [RR 0.50, CI 0.40–0.63, I^2^ 88.9%, Tau^2^ 0.0608, p = <0.001] after receiving the booster dose. The decline in the risk of having Anti-HBs Titer ≤ 10 mIU/ml was variable across all studies, ranging from 17% to 62%. The decline in risk was highest for the study by Bruce *et al*. (2011) and Qian Wu *et al*. (2010) details are shown in Fig. 2.

**Figure 3:**
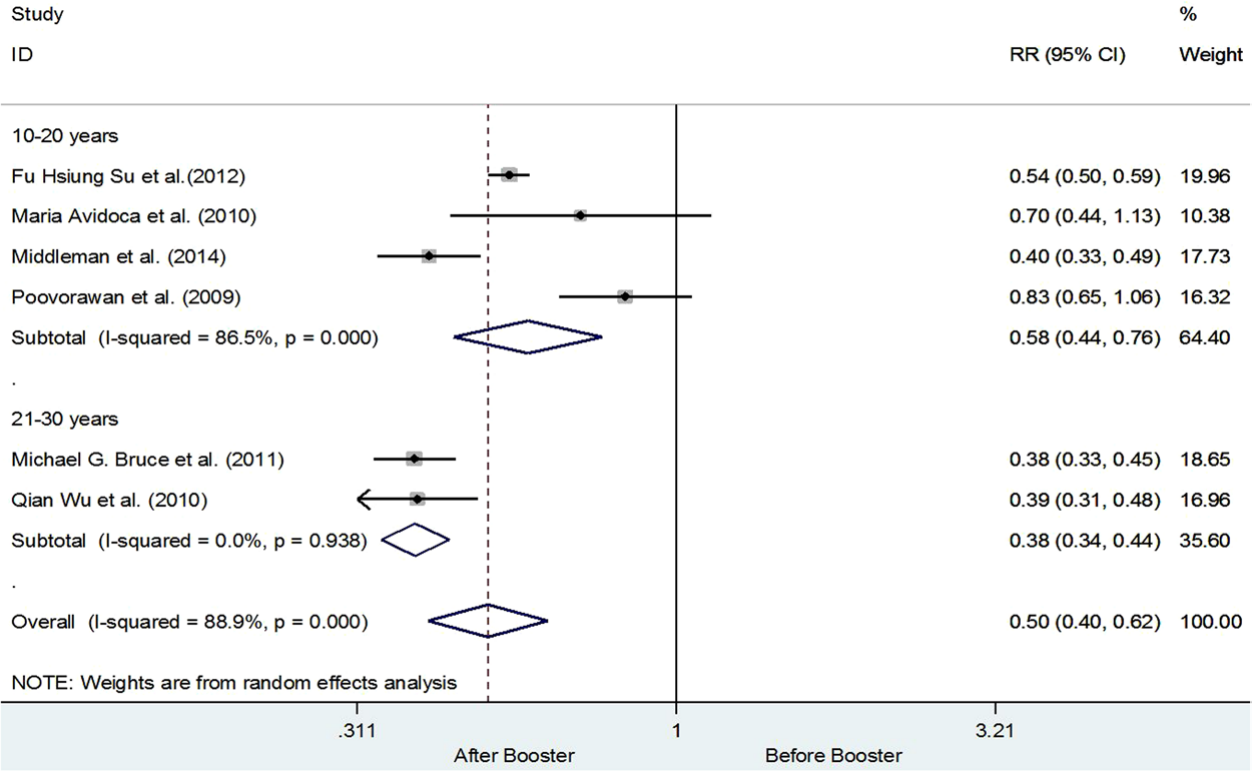
Number of subjects with Anti-HBs Titer ≥ 10 mIU/ml before and after the booster dose using age as the grouping variable.

**Figure 4:**
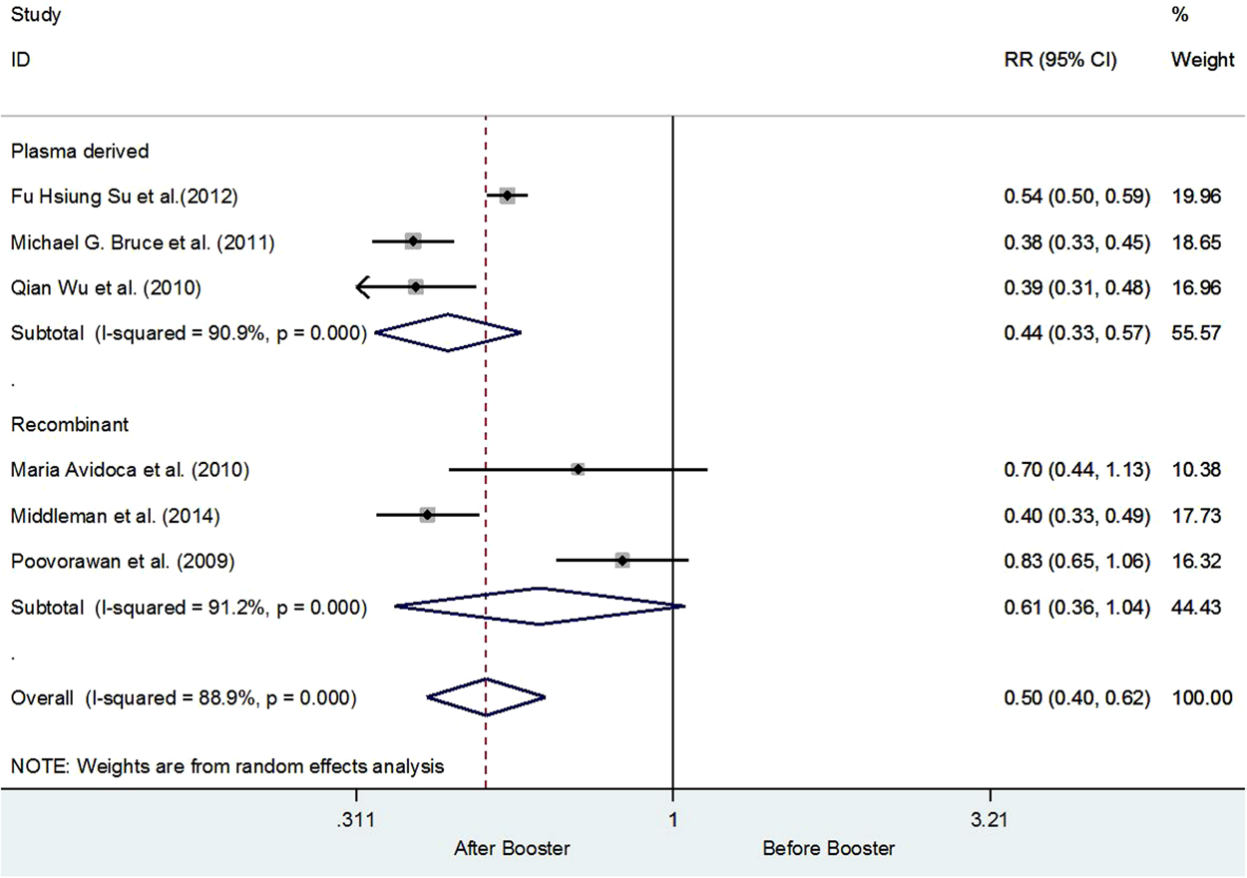
Anti-HBs Titer ≥ 10 mIU/ml before and after the booster dose using types of vaccines as a grouping variable.

Keeping in view the heterogeneity subgroups analysis was performed using age group 10–20 years and 21–30 years respectively. Four studies have recruited subjects from the age of 10–20 years upon receiving the booster dose^20,22,25,26^. While only two studies were from the age group 21–30 years^8,19^. Upon performing the sub-group analysis it was revealed that the overall risk of having Anti-HBs Titer ≤ 10 mIU/ml was reduced up to 42% among the subjects age 10–20 years (0.0.58 [0.44, 0.76]). However, heterogeneity was still 86.5%, while for the age 21–30 years were observed to have 62% decline in the risk of having Anti-HBs Titer ≤ 10 mIU/ml (0.38 [0.34, 0.44]; I^2^ = 0.0%, p = 0.938), In addition, all the studies from the subgroup of 10–20 years of age have adapted a 12 month vaccination schedule in comparison to the 21–30 years of age group who adapted a 6 month vaccination schedule. Details are shown in Fig. 3. Furthermore, it was also observed that the risk of having titer level less than 10 mIU/ml was 39% (RR 0.61 CI 95% 0.36–1.04) less in the recombinant vaccines than the plasma derived vaccines. It is observed that the risk of having titer level less than 10 mIU/ml for plasma derived vaccines were to be 56% [0.44, CI 0.33–0.57, I^2^ 90.9%, p = <0.001]. Details are shown in the Fig. 4.

## Discussion

The current systematic review is perhaps the first to explore the dosing frequency impact on the anti-HBs antibody titer. Anti-HBs antibody titer ≥ 10 mIU/ml and response to booster dose are considered as protection markers against Hepatitis-B infection. Overall, results from the meta-analysis have revealed that the risk of Anti-HBs Titer ≤ 10 mIU/ml reduced by 50%. In addition it was observed that likelihood of the having Anti-HBs Titer ≤ 10 mIU/ml reduced by 42% among the subjects age 10–20 years (0.58 [0.44, 0.76]. Furthermore, with the use of plasma derived vaccines the risk of having Anti-HBs Titer ≤ 10 mIU/ml reduced by 56%. In the studies referred above the protective antibody concentration decreases with time since primary vaccination and booster dose is required to keep antibody titer above seroprotective level. A decrease in anamnestic response has been observed after twenty years of primary vaccination. The response to booster dose decreases with age. This decrease in vaccine protection level is accelerated by vaccination with lower vaccine dose than currently recommended dose at the time of primary vaccination. Therefore without the administration of booster dose the duration of protection in fully vaccinated individuals remains under question.

Data studied for review reveals that after two years of primary vaccination more than 95% of individuals were seroprotected with anti-HBs level ≥ 10 mIU/ml. The level of protection continue to decrease with passage of time as after five years of primary vaccination only 80–90% individuals were found to be seroprotected with anti-HBs level more than 10 mIU/ml. This protection level drops further to 70% ten years after primary vaccination with Hepatitis-B vaccine. Booster dose presents viral challenge in previously immunized individuals which result in spontaneous rise in anti-HBs antibody level due to immunological memory. This rise in antibody level suggests that there is no need for booster dose administration. From the data it is evident that no booster dose is required until 10 years after primary vaccination with Hepatitis-B vaccine.

Limited data is available on duration of protection in adults after the primary vaccination during infancy. Several studies in high risk population show the persistence of immunological memory for as much as 12 years after primary vaccination. Studies on duration of action of Hepatitis-B vaccine is important for healthcare authorities in order to plan for the immunization programs and to make booster dose policy. Lower dose of vaccine and the gap between two consecutive doses seem to be contributing factors in the duration of protection of Hepatitis-B vaccine. The individuals who received the primary vaccination during infancy are better protected. The differences between recombinant vaccine and plasma derived vaccine are also under debate in many countries which require further investigations. No connection is found between the endemic status of the region and duration of protection. The persistence of vaccine immunity requires further studies because large scale seroepidemiological studies in adults are still not been conducted, who are born after the implementation of Hepatitis-B vaccination program. The studies in different ethnic populations are required to determine that same observations are replicable in other ethnic populations as well.

## Limitations

A higher heterogeneity among the studies is one of the main issues that should be kept in view while interpreting the results of the meta-analysis. The heterogeneity among the studies was not statistical and can be due to the clinical and demographics related factors among the participants from the included studies. Secondly we did not investigate the data on duration of protection of Hepatitis-B vaccine in children born with Hepatitis B antigen carrier mothers. The effects of under dose of vaccine and gap between the last dose and the preceding dose during primary vaccination should also be studied for the duration of protection of Hepatitis-B vaccine. Finally the entire analysis was performed using published data, and due to variability of data reporting among the studies further analysis to estimate the dose response relationship was not possible to estimate.

## Conclusion

Results have revealed that risk of having Anti-HBs Titer ≤ 10 mIU/ml reduced by 50% after receiving the booster dose. Use of plasma derived vaccines and participant age of 21–30 years were identified to be the other two factors resulting in higher Anti-HBs titer.

## Recommendations

The protection of an individual remains under question without the administration of booster dose 12 years after primary vaccination during infancy. After this period a booster dose should be administered to check the persistence of immune memory. If the anti-HBS tier remains less than 10 mIU/ml even after the administration of booster dose, revaccination should be done.
